# Molecular characterization of vancomycin-resistant Enterococcus faecium isolates from two German hospitals

**DOI:** 10.3205/dgkh000384

**Published:** 2021-03-17

**Authors:** Laura Nürnberger, Dirk Schmidt, Tobias Szumlanski, Lisa Kirchhoff, Birgit Ross, Jörg Steinmann, Peter-Michael Rath

**Affiliations:** 1 Institute of Medical Microbiology, University Hospital Essen, University of Duisburg-Essen, Essen, Germany; 2 Institute for Clinical Hygiene, Medical Microbiology and Clinical Infectiology, Paracelsus Medical University, Nuremberg Hospital, Nuremberg, Germany; 3 Department of Hospital Hygiene, University Hospital Essen, University of Duisburg-Essen, Essen, Germany

**Keywords:** VRE, MLST, PFGE, molecular epidemiology

## Abstract

**Introduction**: Vancomycin-resistant *Enterococcus* *faecium* accounts for around 10–23% of nosocomial enterococcal infections and constitutes a relevant therapeutic problem due to its limited susceptibility to antibiotics. The resistance towards glycopeptide antibiotics is mediated by the so-called van genes. Currently, the most common resistance type in Germany is the *vanB*-type. Little data are available on the molecular epidemiology in Germany. Therefore, an epidemiological typing of *Enterococcus* *faecium* isolates with vanB-type resistance from two German hospitals in Essen and Nuremberg was performed. Two outbreaks and 104 sporadic cases were investigated.

**Methods:** All 128 isolates with *vanB*-type resistance were collected between 2011–2012 and 2017–2018. They were characterized using multilocus sequence typing (MLST) and pulsed-field gel electrophoresis (PFGE).

**Results:** ST 117 was the most common sequence type (ST) in both hospitals, especially since 2017. PFGE divided the isolates of this study into 68 PFGE types and showed a broad genetic diversity. Two epidemiologically assumed in-hospital outbreaks were genetically confirmed. Apart from that, in-hospital transmissions were rare events.

**Conclusion:** The results obtained by MLST confirmed the previously described allocation of STs in Germany. PFGE showed a broad genetic diversity of *vanB* VRE between the two hospitals and also within each hospital. In-hospital transmissions were rare, but outbreaks did occur. Our data supports the strategy to screen and isolate patients in transmission events in order to detect monoclonality indicating a common source or hygiene mismanagement.

## Zusammenfassung

**Einleitung:** Vancomycin-resistente *Enterococcus faecium* verursachen etwa 10–23% nosokomialer Infektionen durch Enterokokken. Bedingt durch die eingeschränkte Empfindlichkeit gegenüber Antibiotika stellen sie ein erhebliches therapeutisches Problem dar. Die Resistenz gegen Glykopeptid-Antibiotika wird genetisch terminiert durch van-Gene. Gegenwärtig ist der häufigste Typ in Deutschland der *vanB*-Typ mit phänotypischer Resistenz gegen Vancomycin, aber nicht Teicoplanin. Aus Deutschland liegen nur wenige Daten zur molekularen Epidemiologie vor. Daher wurden *E. faecium*-Isolate mit *vanB*-Resistenz mit Herkunft aus zwei Ausbrüchen und 104 sporadischen Fällen molekulargenetisch typisiert.

**Methoden:** Es wurden 128 Isolate mit *vanB*-Resistenz aus den Jahren 2011/12 und 2017/18 mittels Multilokus-Sequenz-Analyse (MLST) und Pulsfeld-Gelelektrophorese (PFGE) typisiert.

**Ergebnisse:** ST117 war der häufigste Sequenztyp (ST) in beiden Krankenhäusern. Mittel PFGE wurden die Isolate in 68 PFGE-Typen unterteilt. Zwei bereits epidemiologisch vermutete Ausbrüche konnten bestätigt werden. Davon abgesehen waren krankenhaus-interne Übertragungen selten.

**Schlussfolgerungen:** Die Ergebnisse der MLST bestätigen die Verteilung von STs in Deutschland. Die PFGE zeigte eine große genetische Diversität von *VanB* positiven *E. faecium*-Isolaten sowohl zwischen den beiden Krankenhäusern wie auch innerhalb der Häuser. Übertragungen im Krankenhaus scheinen selten zu sein, obwohl Ausbrüche vorkommen. Unsere Daten unterstützen die Strategie, Patienten zu screenen und zu isolieren bei Verdacht auf Vorliegen eines Ausbruchs, um Monoklonalität als Hinweis auf Übertragungen und damit Hygienefehler aufzuzeigen.

## Introduction

Enterococci are gram-positive, catalase-negative and facultative anaerobic opportunistic bacteria that can be found as a part of the gastrointestinal microbiota of humans [1], [2]. They can be especially relevant, or pathogenic, for immunocompromised patients, causing different infections [3]. In 2016, a national point prevalence survey reported that 12.6% of the included nosocomial infections were caused by enterococci [4]. Similar to other countries, an increasing rate of resistance to vancomycin (VRE) has been reported for Germany [4].

The most clinically relevant *Enterococcus* species is *Enterococcus* *faecium* (*E. faecium*) [2], [5], being the most frequently resistant species against glycopeptides [6], which also causesmost of the detected enterococcal nosocomial infections [7]. The most common resistance type of *E. faecium* in Germany is *vanB* (phenotypic resistance to vancomycin only) followed by *vanA* (phenotypic resistance to vancomycin and teicoplanin) [8].

In this study, the molecular epidemiology of invasive VRE isolates with the *vanB* phenotype from two German hospitals was analysed. The isolates were characterised by two molecular typing methods to investigate previous outbreaks, and understand possible transmission origins as well as further transmission routes.

## Materials and methods

### Vancomycin resistant enterococcal isolates

Of 146 samples, 128 isolates were *vanB*-positive and belonged to the species *E. faecium*, meeting the inclusion criteria for this study. The 128 clinical isolates were collected in the years 2011–2013 and 2017–2018 at the University Hospital Essen (N=81) and in 2018 at the Hospital Nuremberg (N=47), Germany. In brief, 41% of the isolates were retrieved from patients in intensive care units in both hospitals, 8% were derived from the perinatal centre (outbreak cluster) of the University Hospital Essen and the remaining 51% from units of the other various departments. The isolates were derived from different clinical material. The majority were isolated from rectal swabs, blood cultures (N=20) and urine. No duplicate isolates from a single patient were included. Isolates were identified using matrix-assisted laser desorption/ionization time-of-flight mass spectrometry (MALDI-TOF-MS). They were conserved in cryotubes at –20°C and cultivated on Columbia agar with sheep blood (Oxoid, Wesel, Germany) overnight at 36°C before DNA extraction.

### DNA extraction 

For DNA extraction, the Maxwell16 (Promega, Madison, Wisconsin, USA) was used according to the manufacturer’s instructions [9].

### PCR determination of species and glycopeptide resistance genotype

Species and resistance genes were determined using a specific PCR, as previously described by Dutka-Malen et al. [10]. Isolates that were not found to be *vanB*-positive *E. faecium* were excluded.

### Multilocus sequence typing (MLST)

MLST was carried out with a set of primers amplifying the seven housekeeping genes *adk, atpA, ddl, gyd, gdh, purK, *and* pstS*, according to procedures described by Homan et al. [11]. The obtained sequences were then downloaded from the web page of the LGC genomics shop (https://shop.lgcgenomics.com/) 

and inserted into the MLST database (https://pubmlst.org/bigsdb?db=pubmlst_efaecium_isolates) to determine the sequence types (ST) of the isolates. A phylogenetic analysis was performed with PHYLOViZ software (http://www.phyloviz.net/) using the goeBURST algorithm.

### Pulsed-field gel electrophoresis (PFGE) 

All isolates were characterised by PFGE using the technology from Bio-Rad Laboratories (Hercules, California, USA), based on protocols by Miranda et al. [12]. Briefly, the bacteria were embedded in agarose plugs before the cell walls were lysed and the bacterial genome was digested with the restriction enzyme *SmaI*. The resulting fragments were separated in a 1% agarose gel applying an alternating electric field with 6 V of electric tension at an angle of 120°. The pulse times were 5 s for 4 h, 10 s for 10 h and 25 s for 10 h. The DNA patterns in the gels were stained with ethidium bromide and photo documented with a BioVision device (Vilter, Cudahy, USA). The banding patterns were analysed with CLIQS 1D Pro 1.3.063 software from TotalLab (settings: Jaccard and WPMGA) and visual inspection according to the criteria described by Tenover et al. for outbreaks of *Staphylococcus aureus* [13].

## Results

From a total of 146 initially analysed enterococcal isolates, 18 were rejected after PCR because they did not meet the inclusion criteria (*E. faecium* and *vanB*-positive). Overall, 128 isolates remained for characterisation with MLST and PFGE.

In MLST, the isolates could be assigned to one of a total of 15 detected STs. For some combinations of the seven alleles, no ST was deposited in the database, and when only 6 alleles were included, there was a variety of possible STs. To nevertheless include these isolates in the analysis, they were named after a one-variant ST and marked with an apostrophe (e.g., ST 117’). Furthermore, five isolates were assigned to two different STs, because in a few cases, the two alleles for one gene differed from each other. If both allele combinations were present in the database, the isolates were named after both (e.g., ST 1,301/80). Overall, 15 different STs were distinguished (Table 1 (Tab. 1)).

The most common ST, both overall (43.8%) as well as when Essen (48.2%) and Nuremberg (36.2%) were investigated separately, was ST 117, comprising a total 56 isolates. ST 192 was the most common ST in 2011 and 2012 and the second most common ST overall, with 32 isolates (25%). Due to a lack of isolates collected between 2012 and 2017, evidence cannot be given about that time period. However, it can be stated that ST 192 did not appear again after 2017. Twelve isolates had the ST 80 (9.4%). While only one isolate (0.8%) was found to belong to this ST in Essen, in Nuremberg, there were eleven isolates (23.4%). Considering only the most recent distribution of STs (since 2017), the domination of ST 117 becomes even clearer. In total, 62.4% of all isolates belong to that ST, followed by ST 80 (14.1%) and ST 117’ (8.2%).

**Figure 1:**
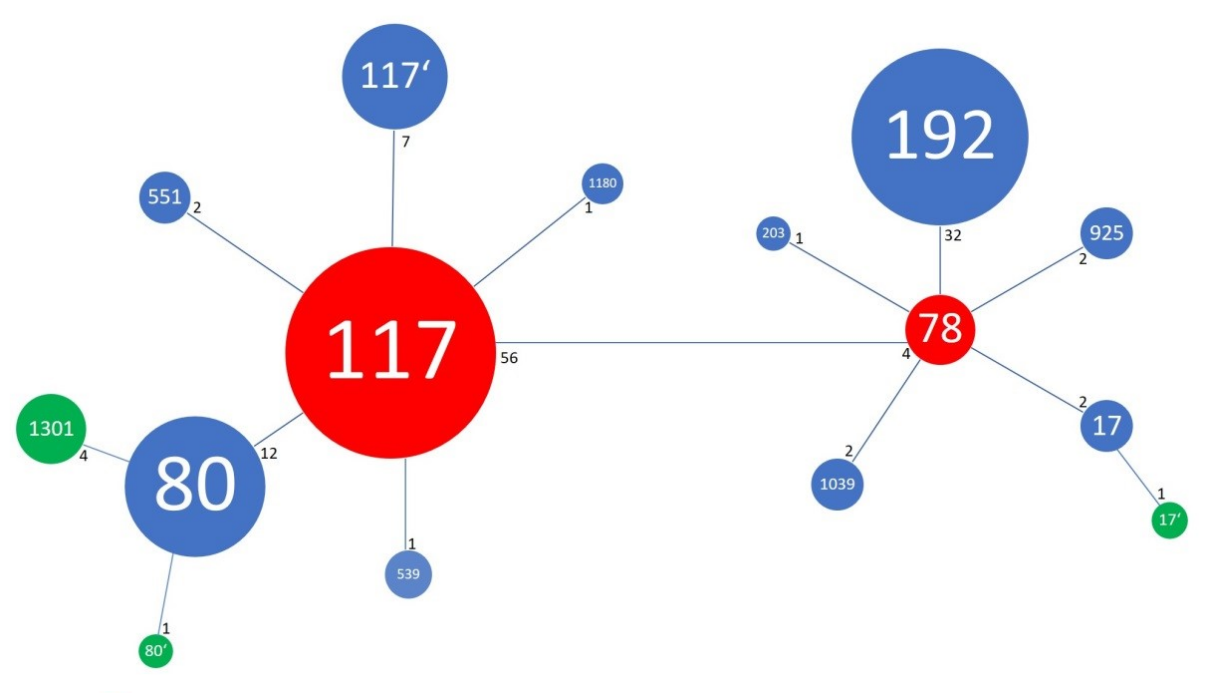
Sequence types (STs) with their allelic differences after goeBURST analysis (PHYLOViZ) A blue line between the STs means one allelic difference (blue circles), two lines two allelic differences (green circles) to the two initial isolates (red circles). The small black numbers are the number of isolates found with that ST. The size of the circles corresponds with the number of isolates.

**Figure 2:**
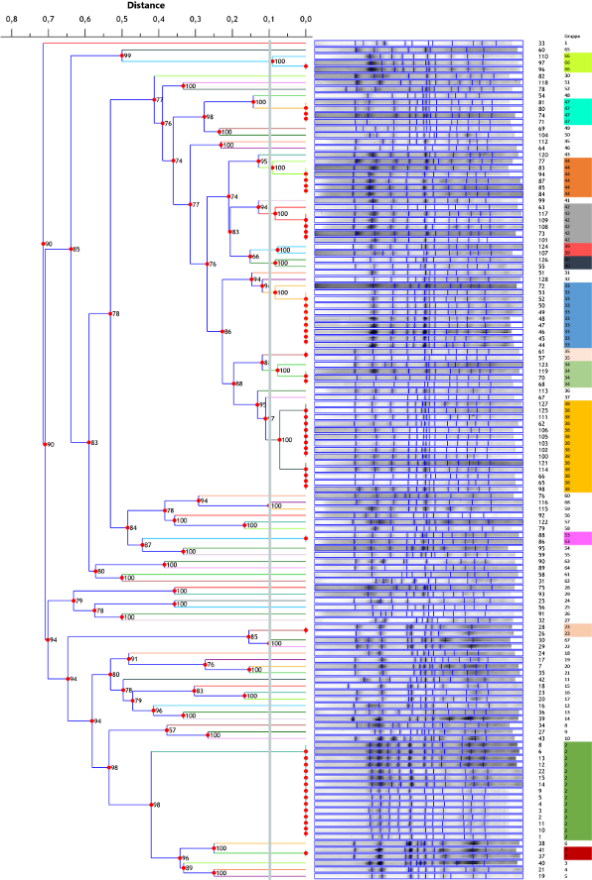
Dendrogram of the 128 isolates after PFGE with *SmaI* restriction The scale shows the similarity between the isolates. The shorter the distance, the greater the degree of relationship. The branches of the isolates that split on the right side of the grey line at 90% belong to one PFGE type (group).

After cluster analysis with the goeBURST algorithm (PHYLOViZ software), the distribution of STs was depicted in a tree diagram (Figure 1 (Fig. 1)). The isolates cluster in a large group with two sub-groups around the two initial isolates ST 117 and ST 192, only differing from these in not more than two of the seven housekeeping genes. In this group, a distinction can be made between one MLST pool from Essen and one from Nuremberg (Table 1 (Tab. 1)). Only the isolates ST 117 and ST 80 were found in both hospitals, although only one isolate from Essen could be assigned to ST 80. All other STs were either found solely in Essen (ST 192, ST 78, ST 1,039/192, ST 925, ST 1,180/117) or solely in Nuremberg (ST 1,301/80, ST 551, ST 17, ST 203, ST 539, ST 117‘, ST 17‘, ST 80‘). After analysis of the PFGE gels, a dendrogram indicating genetic relationship was generated (Figure 2 (Fig. 2)). The cut-off line is at 90%, separating the 128 isolates into 68 PFGE types that differ in no more than one band.

The largest fraction of the isolates (N=15, 12%) belongs to PFGE type 2. All 15 isolates were found on cardiological units of the University Hospital Essen between May and July 2011, with twelve of them on the two intensive care units. Overall, 14 isolates showed a banding pattern that was designated PFGE type 38. Three of those isolates originated from Essen between June and July 2018, and the remaining eleven isolates from various units of the Nuremberg hospital between August and December 2018. With ten isolates showing that banding pattern, PFGE type 33 was identified as the third most common PFGE type. Nine of the ten isolates were found in December 2017 on the intensive care unit and the general neonatology ward of the University Hospital Essen in rectal swabs. The tenth isolate was from the West German Tumour Centre in Essen, where it was also found in a rectal swab seven months later.

In most of the cases, a differentiation between PFGE types from Essen and Nuremberg has been detected. Only five of the 68 PFGE types were found in both hospitals: PFGE type 34, 38, 40, 42 and 44. All the other PFGE types were limited to one facility.

When comparing the classification of the isolates into STs by MLST and PFGE types, they mostly agree with each other. Most isolates of one ST could be divided into different PFGE types, with the following exceptions. Some PFGE types contained isolates with different STs. 14 isolates of PFGE type 2 had ST 192 and one isolate ST 1,039/192. Isolates of PFGE type 34 belonged to three different STs: Two had ST 117, one ST 80 and one ST 539. For PFGE type 35, one isolate had ST 117 and the other one ST 1,180/117. The 14 isolates of PFGE type 38 were allocated to three STs: Ten isolates had ST 17, three ST 117’ and one ST 1,301/80. One isolate with ST 80 and one with ST 1,301/80 belonged to PFGE type 39. PFGE type 42 contained four isolates, two with ST 80, one with ST 1,301/80 and one with ST 117. It was possible to assign the six isolates of PFGE type 44 to ST 80, except for one which belonged to ST 117. PFGE type 66 had two isolates with ST 17 and one with ST 17’.

## Discussion

The 128 *vanB*-positive *E. faecium* isolates from Essen and Nuremberg were divided into 15 STs by MLST analysis. After phylogenetic analysis with the goeBURST algorithm, the two initial ST 117 and 78 could be determined. The initial isolates probably had a survival advantage in the particular environment, and therefore they could easily disperse and proliferate [14]. With time, some of the seven housekeeping genes changed through mutation, which led to new STs that still belonged to the same group [14]. The isolates from Essen and Nuremberg differ for the most part in their STs, which leads to the assumption that the isolates developed and adapted differently due to their different geographical origin.

The 15 STs found in this study all belong to a clonal complex 17 (CC 17) [14], a group of VRE isolates that is responsible for nosocomial infections worldwide [15]. This subpopulation has been shown to be particularly pathogenic [16]. CC 17 is characterised by resistance to ampicillin [15], the *purK* allele 1 [17] and a pathogenicity island linked to the esp virulence gene [18]. In this study, 43 isolates derived from the hospital in Essen between 2011 and 2012, with 32 of these belonging to ST 192. This confirms what the Robert Koch Institute (RKI) described for this time period, with ST 192 being the most prevalent ST detected in Germany with 33% [19]. ST 117 was the most common ST overall in this study, both in Essen and Nuremberg and especially currently. This also is in line with the situation shown by the RKI in the last few years: in 2015 and 2016, ST 117 was the most frequently detected ST of the received isolates, followed by ST 203 and ST 80 [7]. In this study, ST 80 was overall the third-most and in Nuremberg the second-most frequently found ST. In a recently published study, ST 80 and ST 117 were the most common clonal groups in eight hospitals in Bavaria, Germany [20].

The 15 isolates belonging to PFGE type 2 and nine of ten isolates of PFGE type 33 were found in a short period of time and in a small area in the Essen hospital. This confirms the two VRE outbreaks in the cardiology department in 2011 and in the perinatal centre in 2017. In both outbreaks, most isolates only colonised patients’ intestines. There were three bloodstream infections in the 2011 outbreak and one shunt-meningitis in the 2017 outbreak. Both outbreaks were terminated by extensive hygienic measures, such as patient screening, isolation, intensive surface cleaning and closing of the ward (personal communication, Peter-Michael Rath). The tenth isolate of PFGE type 33 was found seven months later in the West German Tumour Centre, which is spatially separate from the outbreak ward. However, complex transmission paths of VRE between different units were previously described by Raven et al. [21].

Some PFGE types were found in both Essen and Nuremberg. It might be possible that a clone spread over this distance. Other studies already described such cross-regional transmissions of PFGE types [1], [7]. However, due to the distance of approximately 500 km, the hospitals have no noticeable exchange of patients or employees. 

A comparison of results from the two methods shows both conformity and disagreements. PFGE gives a more accurate result (68 PFGE types) than does MLST (15 STs). This confirms the proposition that PFGE has a higher discriminatory power than does MLST [22], [23]. However, not all assignments of ST and PFGE types agree with each other. Some PFGE types include isolates with different STs. Some of the discrepancies can be explained by the fact that the isolates which were assigned to two different STs due to two different alleles for one gene were named after both STs and not just assigned to the more common ST. Looking, for example, at PFGE type 2, it is apparent that ST 1039/192 also includes ST 192; therefore, all isolates belong to that ST. The isolates belonging to PFGE type 33 that caused the outbreak in the perinatal centre in 2017 were also analysed by whole genome sequencing (WGS), using the Illumina new genome sequencing technology. The outbreak samples showed an extremely close relationship, differing in only a few variant positions, while three non-VRE isolates could be easily distinguished from them at several hundred positions (publication in preparation). Thus, WGS confirmed the PFGE results.

In conclusion, MLST separated the isolates into a few closely related STs, whereas PFGE showed a broad genetic diversity not only between the two hospitals in Essen and Nuremberg but also within them. Two outbreaks in the hospital in Essen were already epidemiologically recognised by the sudden appearance of VRE in clinical material of numerous patients. Apart from these outbreaks, transmission of VRE within the hospital occur more rarely than assumed. It is more likely that many of the patients acquire their own strain of VRE outside the hospital and then carry it along when hospitalised. Given the well-known low pathogenicity of enterococci for most patient groups (except hemato-oncological patients), our data support the strategy of screening and isolating patients only in outbreaks in order to detect monoclonality which indicates hygienic failures.

## Notes

### Competing interests

The authors declare that they have no competing interests.

### Acknowledgements 

We thank Dr. Ludger Klein-Hitpass (Institute of Cell Biology, University Hospital Essen) for providing the results from WGS.

### Compliance with Ethical Standards

Isolates were analysed anonymously in a retrospective manner. Ethical approval and informed consent were not required. 

### Availability of data and material

The datasets generated and analysed during the current study are either included in this published article or available from the corresponding author on request.

### Funding

Not applicable 

## Figures and Tables

**Table 1:**
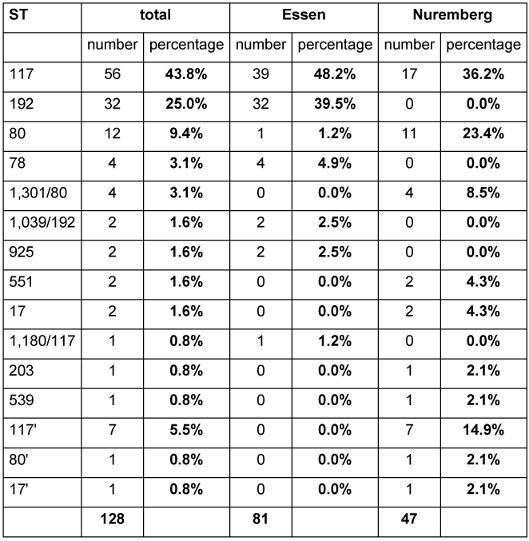
Distribution of sequence types (ST) generated by MLST Overall, 15 different STs could be distinguished. The table shows the total number of isolates belonging to each ST as well as the allocation of STs in the two hospitals in Essen and Nuremberg.
